# Implications and ramifications of using the CDC tier 1 genetic screening concept in East Asian populations

**DOI:** 10.1016/j.gimo.2026.104410

**Published:** 2026-06-06

**Authors:** Kuang-Huan Cheng, Ralph Kirby, Ming-Wei Lin, Chen-Chi Wu, Chih-Cheng Hsu, Shih-Feng Tsai

**Affiliations:** 1 Institute of Molecular Medicine, National Tsing Hua University, Hsinchu, Taiwan; 2 Institute of Molecular and Genomic Medicine, National Health Research Institutes, Miaoli, Taiwan; 3 Institute of Genetics, National Yang Ming Chiao Tung University, Taipei, Taiwan; 4 Institute of Public Health, National Yang Ming Chiao Tung University, Taipei, Taiwan; 5 Department of Otolaryngology, National Taiwan University College of Medicine, Taipei, Taiwan; 6 Department of Medical Research, National Taiwan University Hospital Hsin-Chu Branch, Hsinchu, Taiwan; 7 Institute of Population Health Sciences, National Health Research Institutes, Miaoli, Taiwan; 8 National Center for Geriatrics and Welfare, National Health Research Institutes, Yunlin, Taiwan; 9 Department of Health Services Administration, China Medical University, Taichung, Taiwan; 10 Department of Family Medicine, Min-Sheng General Hospital, Taoyuan, Taiwan

**Keywords:** CDC Tier 1 screening, East Asia, Genome sequencing

## Abstract

**Purpose:**

The US Centers for Disease Control and Prevention defined the Centers for Disease Control and Prevention Tier 1 (CDCT1) genomics applications, advocating mass screening for 3 hereditary conditions with significant public health impacts. On this premise, we investigated how genome sequencing (GS) and ethnic differences affect genetic screening in East Asia. Additionally, we incorporated 2 specific genes, *NOTCH3* (HGNC:7883) and *GJB2* (HGNC: 4284), linked to cerebral autosomal dominant arteriopathy with subcortical infarcts and leukoencephalopathy (MIM: 125310) and hereditary hearing loss (MIM: 220290), respectively, into the study design.

**Methods:**

We analyzed GS data from 1454 participants in the Taiwan Biobank (TWB) and 2473 participants from the National Health Research Institute (NHRI). SnpEff, ClinVar, and VarSome were used to identify and annotate pathogenic and likely pathogenic (P/LP) variants in the target genes.

**Results:**

The heterozygote frequencies for P/LP variants in the CDCT1 genes were 2.34% and 1.74% for the TWB and NHRI cohorts, respectively. These heterozygote frequencies were higher than those of the Healthy Nevada Project (1.33%). Familial hypercholesterolemia (MIM: 143890, 144010) accounts for more than 60% of the cases. Individuals (43%) who carried P/LP variants were unaware of their hypercholesterolemia status. Compared with the European and African populations, 7 variants were significantly more frequent in the Taiwanese population (*P* < .001). Notably, the heterozygote frequencies for cerebral autosomal dominant arteriopathy with subcortical infarcts and leukoencephalopathy with *NOTCH3* NC_000019.10:g.15187315G> A p.(Arg544Cys) were 0.75% and 0.89%, and the frequencies of homozygotes for hereditary hearing loss with *GJB2* NC_000019.10:g.15187315G>A p.(Val37Ile) were 0.69% and 1.01%, in the TWB and NHRI cohorts, respectively.

**Conclusion:**

This Asian study underscores the implications of CDCT1 screening and the significance of including population-specific variants. Using a GS approach allows for broader detection of actionable diseases, advancing population-based preventive medicine.

## Introduction

The US Centers for Disease Control and Prevention Tier 1 (CDCT1) genomics applications have the potential to identify individuals at increased risk of suffering from several specific genetic diseases, as well as to allow surveillance at a younger age by targeting families at risk. Follow-up actions for heterozygotes of 3 hereditary conditions, hereditary breast and ovarian cancer syndrome (HBOC) (MIM: 604370, 612555, 620442), Lynch syndrome (LS) (MIM: 120435, 609310, 614337, 614350, 613244), and familial hypercholesterolemia (FH) (MIM: 143890, 144010, 603776, 603813) are recommended in the CDC evidence-based guidelines to positively reduce risk and provide benefits to public health care systems.^1^ A population genomics study by Haverfield et al^2^ involving 10,478 individuals observed a 2% prevalence of pathogenic/likely pathogenic (P/LP) variants while using the CDCT1 gene panel. A recent Healthy Nevada Project (HNP) study found a 1.33% combined heterozygote frequency for P/LP genetic variants; in addition, 90% of the risk heterozygotes would not have been identified during routine care.^3^ Furthermore, another study in Australia offered 10,000 preventive DNA screenings for individuals between 18 and 40 years of age through the Australian public health care system.^4^ Thus, CDCT1 genomics applications are promising for identifying actionable genetic risk factors and facilitating early detection and prevention of genetic diseases.

Although the HNP screening was effective, more than 90% of the participants were of either White or Hispanic-Latino descent, and only 3% were of Asian ancestry.^3^ This stark disparity underscores the urgent need for population genetic studies in Asian populations. Our investigation into the usefulness of CDCT1 genes for screenings in Asia by interrogating the P/LP variants of these genes in Asian genomic databases is a crucial step toward addressing this need.

Expanding the CDCT1 approach, 2 prevalent genetic diseases in Taiwan, cerebral autosomal dominant arteriopathy with subcortical infarcts and leukoencephalopathy (CADASIL) (MIM: 125310) and hereditary hearing loss (HHL) (MIM: 220290), were considered as potential targets for population screening. CADASIL is an autosomal dominant disease involving the compromise of small vessel integrity, with its characteristic manifestations being recurrent ischemic events, subcortical dementia, mood disturbance, and migraine with aura.^5^ Nearly 0.88% of the people in Taiwan carry the *NOTCH3* NC_000019.10:g.15187315G>A p.(Arg544Cys) variant.^6^ Among 112 patients with CADASIL from 95 families in Taiwan, *NOTCH3* NC_000019.10:g.15187315G>A p.(Arg544Cys) was the most common variant, accounting for 70.5% of the pedigrees.^7^ The other disease, HHL, affects approximately 1 in 1000 infants, and *GJB2* mutants are responsible for 21.7% of hearing loss in 420 families from a Taiwanese cohort, with the most common variants being *GJB2* NC_000013.11:g.20189473C>T p.(Val37Ile) and NC_000013.11:g.20189349del p.(Leu79fs).^8^ Adding these 2 genes to a genetic screening program in Taiwan will identify possible heterozygotes in advance and will help the family members be aware of their disease risks.

Before implementing CDCT1 genetic screening in Taiwan, we sought to ascertain the potential outcomes of adopting a more comprehensive methodology, namely, genome sequencing (GS). We used genome sequences obtained from previous studies and ongoing projects to estimate the prevalence of positive findings. Our results corroborate the general principle of CDCT1 genetic screening and the feasibility of a sequencing-based approach. Furthermore, we identified novel variants that were significant in East Asian (EAS) populations.

## Materials and Methods

### Recruitment and enrollment from cohorts

The National Health Research Institutes (NHRI) cohort is a comprehensive human participant research study based in Taiwan that explores healthy aging and various diseases, including cancer. The Healthy Aging Longitudinal Study in Taiwan (HALST) cohort recruited individuals aged ≥55 years. The exploration of healthy aging consisted of home interviews and hospital-based clinical examinations every 5 years from 2009 to the present.^9^ Among the 5664 participants, 1024 have undergone GS and have complete electronic health records. Epilepsy, Fabry disease, obesity, hepatocellular carcinoma, and lung cancer patients were also recruited within the NHRI cohort, and these individuals comprised 1548 GS samples.

Since 2012, the Taiwan Biobank (TWB) has been conducting a prospective study of more than 150,000 individuals aged 20-70 years in Taiwan. TWB developed 2 customized single-nucleotide variant (SNV) arrays with GS, which have been used for genotyping. The study includes 1454 GS samples, various clinical measurements, and information from questionnaires.^10^ Altogether, just more than 4000 GS samples were collected from the NHRI cohort (2572 genomes) and the TWB cohort (1454 genomes). We applied the SnpEff tool to filter high- and moderate-impact variants, which were then searched against 2 databases, ClinVar and VarSome, to pinpoint variants for further scrutiny.

### Genome sequencing

All the participants in the NHRI cohort were sequenced in Taiwan at the NHRI Core Instrument Center using the Illumina NovaSeq 6000 instrument. GS libraries were constructed using the Illumina DNA PCR-Free Library Preparation Kit. Variant calling was performed through the Nvidia Clara Parabricks v4.0.1 germline pipeline^11^ using the reference genome build (version GRCh38). Quality control was performed using the Genome Analysis Toolkit (GATK) Variant Quality Score Recalibration pipeline^12^ to filter out probable artifacts and to exclude third-degree relatives by PLINK 1.9.^13^ Finally, 2473 genomes were retained for our study.

In addition to the NHRI cohort, GS data from the TWB were also incorporated. The TWB includes more than 150,000 participants, most of whom were genotyped using array-based platforms, whereas only a small subset has undergone GS; these sequenced samples are publicly available for research use. Sequencing was performed using both the Thermo Fisher Ion Proton and Illumina platforms, yielding approximately 90 GB per sample with an average depth of 30 ×. The Ion Proton sequencing and initial data processing were conducted by Taiwan Integrated Genomics Solutions Co, Ltd, whereas the Illumina sequencing was carried out by Genomics BioSci & Tech Co, Ltd.^10^^,^^14^ To ensure comparability across datasets, the same variant-calling, quality-control, and relatedness-filtering procedures described above were also applied to the TWB GS samples.

### Selection of genes for screening

Our candidate genes were integrated into the CDCT1 genomics applications, the Australian Population DNA Screening, INVITAE First Tier Population Screen, and genes prevalent in Asian populations related to CADASIL and HHL.

The CDCT1 genomics application screens for 3 hereditary disease conditions (HBOC, LS, and FH), which comprise the following genes: *BRCA1* (HGNC:1100), *BRCA2* (HGNC:1101), *MLH1* (HGNC:7127), *MSH2* (HGNC:7325), *MSH6* (HGNC:7329), *PMS2* (HGNC:9122), *APOB* (HGNC:603), *LDLR* (HGNC:6547), and *PCSK9* (HGNC:20001).^1^

The Australian Population DNA Screening targets HBOC, LS, and FH and consists of the following genes: *BRCA1*, *BRCA2*, *PALB2* (HGNC:26144), *MLH1*, *MSH2*, *MSH6*, *APOB*, *LDLR*, and *PCSK9*.^4^ The INVITAE First Tier Population Screen was intended to screen individuals for HBOC, LS, and FH. The test comprised the CDCT1 genes and was designed to screen individuals for HBOC, LS, and FH. Additionally, it included the CDCT1 genes, *EPCAM* (HGNC:11529) and *LDLRAP1* (HGNC:18640).^15^

Asian prevalent genes related to CADASIL and HHL were *NOTCH3* and *GJB2*.

Together, we employed a comprehensive screening approach using 14 genes, namely CDCT1, *PALB2*, *EPCAM*, *LDLRAP1*, *NOTCH3*, and *GJB2*.

### Ancestry analysis

To characterize the genetic ancestry of the NHRI and the TWB GS cohorts, we performed principal component analysis (PCA) using genome-wide genotype data. Variants with a call rate <95% or minor allele frequency <1% were excluded. To reduce linkage disequilibrium, SNVs were pruned using PLINK^13^ with a window size of 500 SNVs, a step size of 50 SNVs, and an r^2^ threshold of 0.2. PCA was conducted with the remaining variants using the ‘–pca’ function in PLINK. The 1000 Genomes Project phase 3 populations (EAS, European [EUR], African [AFR], Admixed American, and South Asian) were included as references to infer population structure.^16^

### Classification of the P/LP alleles

All variants were first annotated using SnpEff, a genetic variant annotation and functional effect prediction toolbox. SnpEff annotates and predicts the impact of genetic variants on genes and proteins.^17^ We filtered for variants with high-impact predictions; these are expected to have a high (disruptive) effect on the relevant protein and cause either protein truncation, loss of function, or trigger nonsense-mediated decay. We also filtered for variants that had a moderate impact; these include non-disruptive variants that might change protein effectiveness.^18^ After filtration, the variants with high and moderate impact were interpreted using VarSome and ClinVar.

VarSome (version: 11.12.2) incorporates 140+ data sources and is continuously updated following the American College of Medical Genetics and Genomics guidelines.^19^ The Germline Classifier Verdict uses a point system based on the strength of the evidence provided.^20^ The total score is then compared with thresholds to assign the final verdict:

• P if ≥10
• LP if between 6 and 9 inclusive
• Uncertain significance if between 0 and 5 inclusive
• Likely benign if between −6 and −1 inclusive
• Benign if ≤ −7

ClinVar (version: February 7, 2024) is a database maintained by the US National Center for Biotechnology Information that connects variants with clinically associated phenotypes.^21^

Finally, all P/LP variants of the CDCT1 genes and *PALB2*, *EPCAM*, *LDLRAP1*, *NOTCH3*, and *GJB2* were collected.

### Comparison of allele frequencies across global databases

Allele frequencies of selected variants were compared across diverse cohorts and datasets (Supplemental Table 1). For the Taiwanese cohorts, in addition to the NHRI and TWB GS data, Taipei Veteran General Hospital provided Taiwan Precision Medicine Initiative (*n* = 37,790) array data.^22^ Selected variants from the TWB array data (TWB 1.0 [*n* = 24,670] and TWB 2.0 [*n* = 100,277]) were screened on the TWB website.^14^ Not available variants mean that the variants were not explored.

The SG10K pilot study included 4810 samples, including 2780 Chinese, 903 Malays, and 1127 Indians. Data are available through the SG10K website.^23^^,^^24^

TogoVar integrates allele frequencies derived from Japanese populations and provides annotations for variant interpretation. Selected variants were screened using the ToMMo 54KJPN-SNV/INDEL Allele Frequency Panel (*n* = 54,302) (v20230626r3) and GS data.^25^

The Genome Aggregation Database (gnomAD) v4 provides information regarding the frequency of each allele in a set of 730,947 exome sequences and 76,215 whole genomes from diverse ancestries.^26^

To optimize the relatively high risks associated with CDCT1, *NOTCH3*, and *GJB2* genes, we established allele frequencies of 0.1%, and selected variants were estimated using combined NHRI and TWB GS data. Reference frequencies were obtained from SG10K Chinese, SG10K Malay, TogoVar Japan, gnomAD EAS, EUR (non-Finnish), and AFR cohorts.^23^, ^24^, ^25^, ^26^ Odds ratios (ORs) were calculated to quantify enrichment. For comparisons involving 0 counts in any population (eg, *LDLR* variants were absent in the TogoVar dataset), the Haldane–Anscombe 0.5 continuity correction was applied by adding 0.5 to all cells in the contingency table to produce stable and finite OR estimates. Differences between Taiwanese and reference populations were tested using Fisher Exact Test, with significance defined as *P* < .05.

### Electronic health records and survey data

The HALST dataset consists of medical information and is updated every 5 years.^9^ During each wave, FH heterozygotes are recorded as either taking or not taking cholesterol-lowering medication. The TWB dataset includes 1400 participants’ demographics, clinical characteristics, oral interactive survey data, etc.^10^ VarSome and ClinVar define participants as heterozygotes for HBOC, LS, and FH.

### Statistical analysis

The HALST and TWB datasets were analyzed using an unpaired Mann–Whitney U test. Using the HALST dataset, FH and non-FH heterozygotes were analyzed in waves 1 and 2, and VarSome and ClinVar were used to interpret the results. *P* values represent the results of a 1-sided test. The TWB dataset was analyzed for HBOC, LS, and FH heterozygotes based on clinical measurements and related diseases. The *P* values represent the results of a 2-sided test.

## Results

### Ancestry analysis of the taiwanese cohorts

**Figure 1:**
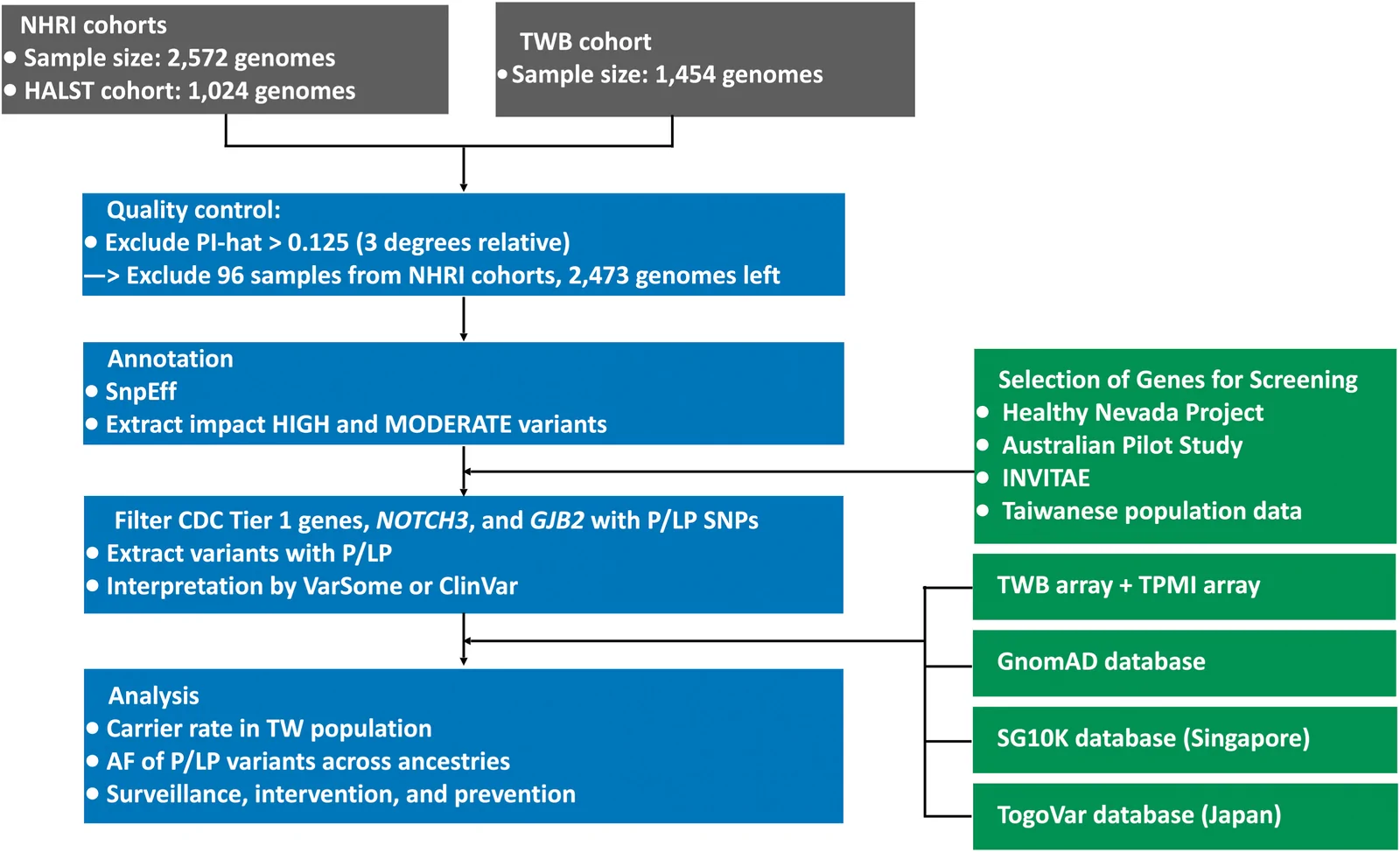
**Identification of significant genetic variants from genome sequencing data and mass-screening design on selected cohorts in Taiwan.** CDC, the Center for Disease Control and Prevention; ClinVar, the clinical variant database; HALST, the Healthy Aging Longitudinal Study in Taiwan; LP, likely pathogenic; NHRI, the National Health Research Institute; P, pathogenic; TW, Taiwan; TWB, the Taiwan Biobank; TPMI, the Taiwan Precision Medicine Initiative.

Based on the workflow shown in Figure 1, the analysis began by integrating the NHRI and TWB cohorts, comprising 2473 and 1454 genomes, respectively, to identify genetic variants in the Taiwanese population. To characterize the population background, we assessed the ethnic composition of all participants from the NHRI cohort and the TWB GS subset. PCA using reference populations from the 1000 Genomes Project showed that all Taiwanese samples formed a single, well-defined genetic cluster within the EAS superpopulation and clustered most closely with Southern Han Chinese when the PCA was restricted to EAS populations (Supplemental Figure 1).

### Heterozygote frequencies of P/LP variants in taiwanese CDCT1 genetic screening

In this study, we searched the available genomic information from Taiwan, taking GS data from the TWB and the NHRI study cohorts. We selected 12 candidate genes based on the HNP, the Australian Pilot Study, and the INVITAE genetic screening approach. These consisted of 3 HBOC-related genes (*BRCA1*, *BRCA2*, and *PALB2*), 5 LS-related genes (*MLH1*, *MSH2*, *MSH6*, *PMS2*, and *EPCAM*), and 4 FH-related genes (*APOB*, *LDLR*, *PCSK9*, and *LDLRAP1*). All the variants were identified and annotated using VarSome and ClinVar. Ninety-nine unique P/LP variants linked to CDCT1 genetic conditions were identified (Supplemental Table 2). The P/LP variants identified by ClinVar for CDCT1 genetic conditions were carried by 34 of 1454 individuals and 43 of 2473 individuals, giving detection rates of 2.34% and 1.74%, for the TWB and NHRI cohorts. These heterozygote frequencies were higher than those observed in the HNP (1.33%), and FH heterozygotes account for approximately 60% of the positive cases (Table 1).

**Table 1:** Individuals with hereditary breast and ovarian cancer syndrome, Lynch syndrome, and familial hypercholesterolemia in the cohorts were heterozygous for a pathogenic/likely pathogenic variant as identified by the clinical variant database and VarSome

Studied Cohorts	Annotation	HBOC				LS						FH					Total
		*BRCA1*	*BRCA2*	*PALB2*	Total	*MLH1*	*MSH2*	*MSH6*	*PMS2*	*EPCAM*	Total	*APOB*	*LDLR*	*PCKS9*	*LDLRAP1*	Total	
Individuals with P/LP variants in TWB																	
TWB (*n* = 1454)	ClinVar	0	4	1	5	0	1	3	4	0	8	7	14	0	0	21	34 (2.34%)
	VarSome	6	10	2	18	7	9	7	9	1	32^a^	12	18	1	0	31	80 (5.50%)^a^
Male (*n* = 717)	ClinVar	0	3	1	4	0	1	1	1	0	3	2	7	0	0	9	16 (2.23%)
	VarSome	3	3	1	7	6	2	3	3	0	14	5	8	1	0	14	35 (4.88%)
Female (*n* = 737)	ClinVar	0	1	0	1	0	0	2	3	0	5	5	7	0	0	12	18 (2.44%)
	VarSome	3	7	1	11	1	7	4	6	1	18^a^	7	10	0	0	17	45 (6.11%)^a^
Individuals with P/LP variants in NHRI cohorts																	
NHRI cohorts (*n* = 2473)	ClinVar	3	2	3	8	0	0	1	1	1	3	6	27	0	0	32^a^	43 (1.74%)
	VarSome	5	16	3	23^a^	14	9	2	3	1	29	17	33	5	0	54^a^	105 (4.25%)^a^
Male (*n* = 1270)	ClinVar	3	1	0	4	0	0	1	1	0	2	4	11	0	0	15	21 (1.65%)
	VarSome	5	6	0	10^a^	5	1	2	1	0	9	8	13	2	0	23	41 (3.23%)^a^
Female (*n* =1203)	ClinVar	0	1	3	4	0	0	0	0	1	1	2	16	0	0	17^a^	22 (1.83%)
	VarSome	0	10	3	13	9	8	0	2	1	20	9	20	3	0	31^a^	64 (5.32%)
Individuals with P/LP variants in HALST cohort																	
HALST (*n* = 1016)	ClinVar	1	0	2	3	0	0	0	0	1	1	1	9	0	0	10	14 (1.38%)
	VarSome	2	6	2	10	5	5	0	1	1	12	3	12	4	0	19	40 (3.94%)^a^
Male (*n* = 443)	ClinVar	1	0	0	1	0	0	0	0	0	0	1	4	0	0	5	6 (1.35%)
	VarSome	2	2	0	4	2	0	0	0	0	2	2	4	2	0	8	13 (2.93%)^a^
Female (*n* = 573)	ClinVar	0	0	2	2	0	0	0	0	1	1	0	5	0	0	5	8 (1.40%)
	VarSome	0	4	2	6	3	5	0	1	1	10	1	8	2	0	11	27 (4.71%)

Values are presented as *n* or *n* (%)

*ClinVar*, the clinical variant database; *FH*, familial hypercholesterolemia; *HALST*, the Healthy Aging Longitudinal Study in Taiwan; *HBOC*, hereditary breast and ovarian cancer syndrome; *LP*, likely pathogenic; *LS*, Lynch syndrome; *NHRI*, the National Health Research Institute; *P*, pathogenic; *TWB*, the Taiwan Biobank.

a Heterozygotes with 2 variants derived from 2 genes. One heterozygote had 2 familial hypercholesterolemia pathogenic/likely pathogenic variants in the National Health Research Institute cohorts annotated by the clinical variant database; 9 heterozygotes had 2 center for disease control and prevention Tier 1 pathogenic/likely pathogenic variants in both the Taiwan Biobank and the National Health Research Institute cohorts annotated by VarSome.

Next, the heterozygote distributions were compared between the ClinVar- and VarSome-annotated results (Table 1). The ClinVar-interpreted heterozygotes for HBOC were distributed across the *BRCA1*, *BRCA2*, and *PALB2* genes, whereas the ClinVar-interpreted heterozygotes for LS were mainly limited to the *MSH2*, *MSH6*, and *PMS2* genes. Similarly, the ClinVar-interpreted heterozygotes for FH were limited to the *APOB* and *LDLR* genes. When annotated using VarSome, the number of heterozygotes increased substantially across all 3 disease categories, consistent with the approximately 2-fold higher heterozygote frequencies observed. VarSome identified more HBOC heterozygotes in each gene (*BRCA1*, *BRCA2*, and *PALB2*), expanded the LS heterozygotes across *MLH1*, *MSH2*, *MSH6*, *PMS2*, and *EPCAM*, and detected additional FH heterozygotes in *APOB* and *LDLR*. Interestingly, *LDLRAP1* heterozygotes were not identified by either approach.

### Expansion of taiwanese CDCT1 genetic screening to include P/LP variants in *NOTCH3* and *GJB2*

We expanded the CDCT1 genetic screening concept by incorporating 2 genes, *NOTCH3* and *GJB2*, associated with CADASIL and HHL in Taiwan. Following the same workflow as CDCT1 screening, we found 29 P/LP variants related to CADASIL and HHL (Supplemental Table 3). For CADASIL, the *NOTCH3* P/LP variants interpreted by ClinVar consist mainly of the *NOTCH3* NC_000019.10:g.15187315G>A p.(Arg544Cys) variant, which was present in 11 individuals (0.75% heterozygote frequency) and 22 individuals (0.89% heterozygote frequency) in the TWB and NHRI cohorts, respectively (Supplemental Table 4). The *GJB2* NC_000013.11:g.20189473C>T p.(Val37Ile) variant accounted for approximately 90% of P/LP *GJB2* variants for HHL. Specifically, 10 (of 1454, 0.69%) and 25 (of 2,473, 1.01%) individuals were identified to have *GJB2* NC_000013.11:g.20189473C>T p.(Val37Ile) homozygous variants, which are likely to result in a significantly higher hearing loss risk.^27^ When combined with the *GJB2* NC_000013.11:g.20189473C>T p.(Val37Ile) heterozygous variant, the heterozygote frequencies substantially increased to 16.64% and 17.02% for the TWB and NHRI cohorts, respectively (Supplemental Table 4). P/LP variants interpreted by VarSome showed slightly higher heterozygote frequencies.

### Seven P/LP variants have higher allele frequencies in the taiwanese population

Within the 99 P/LP variants in CDCT1 genes and 29 P/LP variants in *NOTCH3* and *GJB2* identified, we filtered for allele frequencies higher than 0.1% and identified 7 variants linked to CDCT1 genetic conditions, CADASIL, and HHL (Table 2). Among the 7 identified P/LP variants, classifications were generally consistent between ClinVar and VarSome. Specifically, 3 variants *APOB* NC_000002.12:g.21006289G>A p.(Arg3527Trp), *LDLR* NC_000019.10:g.11102741G>A p.(Asp90Asn), and *LDLR* NC_000019.10:g.11116900C>T p.(His583Tyr) are associated with FH, and the 3 *GJB2* variants were consistently annotated as P/LP by both databases. The *NOTCH3* NC_000019.10:g.15187315G>A p.(Arg544Cys) variant was classified as P/LP by ClinVar and as LP by VarSome.

**Table 2:** Pathogenic/likely pathogenic variants for the CDCT1, *NOTCH3*, and *GJB2* genes with allele frequency >0.1% in the Tiwan biobank and the National Health Research Institutes cohorts

Gene	HGNC Gene ID	Variant	Function	HGVS Descriptions	ClinVar Annotation	VarSome Annotation
*APOB*	603	2-21006289-G-A	missense_variant	NC_000002.12:g.21006289G>A p.(Arg3527Trp)	P/LP	P
*LDLR*	6547	19-11102741-G-A	missense_variant	NC_000019.10:g.11102741G>A p.(Asp90Asn)	P	P
*LDLR*	6547	19-11116900-C-T	missense_variant	NC_000019.10:g.11116900C>T p.(His583Tyr)	P/LP	P
*NOTCH3*	7883	19-15187315-G-A	missense_variant	NC_000019.10:g.15187315G>A p.(Arg544Cys)	P/LP	LP
*GJB2*	4284	13-20189473-C-T	missense_variant	NC_000013.11:g.20189473C>T p.(Val37Ile)	P	P
*GJB2*	4284	13-20189346-AG-A	frameshift_variant	NC_000013.11:g.20189349del p.(Leu79fs)	P	P
*GJB2*	4284	13-20189281-CAT-C	frameshift_variant	NC_000013.11:g.20189282_20189283del p.(His100fs)	P	P

*Note*: The genome build used for analysis is GRCh38 version.

*ClinVar*, the clinical variant database; *HGNC*, HUGO Gene Nomenclature Committee; *HGVS*, Human Genome Variation Society; *LP*, likely pathogenic; *P*, pathogenic.

To validate the clinical relevance of our findings, we assessed concordance with variants in previous research. We compared the variant interpretations with regard to the results from a genetic analysis of a Taiwanese cohort of 750 index patients with clinically diagnosed FH.^28^ Notably, 3 genetic variants *APOB* NC_000002.12:g.21006289G>A p.(Arg3527Trp), *LDLR* NC_000019.10:g.11102741G>A p.(Asp90Asn), and *LDLR* NC_000019.10:g.11116900C>T p.(His583Tyr) were also reported as clinically significant, confirming their relevance in FH pathogenesis, as reported by Huang et al.^28^ Hence, our findings showed consistent alignment with known clinical associations for CADASIL and HHL,^6^, ^7^, ^8^ further supporting the clinical validity and applicability of the variants identified through this expanded screening framework.

### Ethnic differences in the CDCT1, *NOTCH3* and *GJB2* genetic variants across populations

To further elucidate any ethnic specificities in the Taiwanese cohorts, we collected population data related to the frequencies of the 7 P/LP variant alleles from the gnomAD, SG10K, and TogoVar databases (Supplemental Table 1). Pairwise statistical comparisons highlighted distinct population differences. The Taiwanese cohorts showed higher frequencies of 7 specific *APOB*, *LDLR*, *NOTCH3*, and *GJB2* variants compared with EUR and AFR populations (ORs >60, *P* < .001) (Table 3). Among EAS groups, the Taiwanese cohorts most closely resembled the SG10K Chinese population, although differences remained for *GJB2* NC_000013.11:g.20189473C>T p.(Val37Ile) variant (*P* < .01) (Supplemental Table 5).

**Table 3:** Statistical analysis of allele frequencies: Taiwanese cohorts vs European and African the genome aggregation database datasets

Variant (Taiwanese African)	Dataset	OR (95% CI)	*P* Value (Fisher's Exact Test)
*APOB* NC_000002.12:g.21006289G>A p.(Arg3527Trp) (*P* =.00203)	gnomAD European (non-Finnish)	69.64 (38.12-127.2)	< .001
	gnomAD African	98.87 (18.5-528.4)	< .001
*LDLR* NC_000019.10:g.11102741G>A p.(Asp90Asn) (*P* =.00101)	gnomAD European (non-Finnish)	111.61 (52.03-239.4)	< .001
	gnomAD African	219.7 (12.95-3728.8)	< .001
*LDLR* NC_000019.10:g.11116900C>T p.(His583Tyr) (*P* =.00229)	gnomAD European (non-Finnish)	5571.23 (335.70-92459.5)	< .001
	gnomAD African	353.82 (21.32-5872)	< .001
*NOTCH3* NC_000019.10:g.15187315G>A p.(Arg544Cys) (*P* =.004202)	gnomAD European (non-Finnish)	1123.07 (419.69-3005.3)	< .001
	gnomAD African	642.69 (39.37-10490.5)	< .001
*GJB2* NC_000013.11:g.20189473C>T p.(Val37Ile) (*P* =.088872)	gnomAD European (non-Finnish)	110.56 (100.17-122)	< .001
	gnomAD African	131.85 (100.21-173.5)	< .001
*GJB2* NC_000013.11:g.20189349del p.(Leu79fs) (*P* =.005602)	gnomAD European (non-Finnish)	1920.9 (646.93-5703.6)	< .001
	gnomAD African	855.15 (52.66-13887.7)	< .001
*GJB2* NC_000013.11:g.20189282_20189283del p.(His100fs) (*P* =.001019)	gnomAD European (non-Finnish)	365.23 (105.16-1268.4)	< .001
	gnomAD African	162.6 (9.38-2817.5)	< .001

*CI*, confidence interval; *gnomAD*, the genome aggregation database; *OR*, Odds ratio.

Despite general regional similarity, the Taiwanese population diverged from gnomAD EASs for *APOB*, *LDLR*, *NOTCH3* and *GJB2* NC_000013.11:g.20189473C>T p.(Val37Ile) variants (OR: 2.08-4.71, *P* < .001). Comparison with the Japanese population (TogoVar) revealed even more extreme enrichment: *LDLR* NC_000019.10:g.11102741G>A p.(Asp90Asn) and *LDLR* NC_000019.10:g.11116900C>T p.(His583Tyr) (OR: 318.47 and OR: 512.78), *NOTCH3* NC_000019.10:g.15187315G>A p.(Arg544Cys) (OR: 186.06), and *APOB* NC_000002.12:g.21006289G>A p.(Arg3527Trp) (OR: 143.14). Among *GJB2* variants, NC_000013.11:g.20189349del p.(Leu79fs) frequencies were similar to those in SG10K Chinese, Japanese, and gnomAD EAS cohorts but were notably higher than in SG10K Malays (OR: 42.60). Overall, the Taiwanese population exhibits distinct enrichment of several variants, highlighting notable differences from both global (Table 3) and regional (ie, EAS) (Supplemental Table 5) points of view.

### Health care management implications for Taiwan

In total, 1400 TWB individuals provided medical and survey records, and we analyzed these individuals to identify those carrying gene variants associated with cardiovascular disease and metabolic dysfunction. This analysis was conducted using the 2-sided Mann–Whitney U test. As indicated in Supplemental Table 6, genetic variants present in the CDCT1 genes are significantly associated with valvular heart disease, coronary artery disease, cardiomyopathy, hypercholesterolemia, cholesterol levels, and low-density lipoprotein levels in the TWB cohort. A similar approach was applied to FH heterozygotes using the HALST data. Notably, cholesterol and low-density lipoprotein-C levels exhibited no significant impact in the 2 waves interpreted by VarSome and ClinVar (Supplemental Figure 2). Furthermore, HBOC and LS heterozygotes did not show a statistically significant association (*P* > .05) with the presence of relevant cancers or diseases (Supplemental Tables 7 and 8). Nevertheless, more than 42.86% of the individuals who carried a P/LP variant for FH were aware of their hypercholesterolemia (Table 4). Conversely, in the HALST study, only 1 individual (33%) was utilizing a statin during the initial wave of the HALST survey, and this proportion increased to 2 (44%) in the subsequent wave survey (Supplemental Table 9).

**Table 4:** The awareness of familial hypercholesterolemia heterozygotes in the Taiwan biobank cohort (*n* = 1400)

Annotated Tool	FH Heterozygotes	Awareness Group	Unawareness Group
VarSome	28	12 (42.86%)	16 (57.14%)
ClinVar	14	8 (57.14%)	6 (42.86%)

Values are presented as *n* or *n* (%).

*ClinVar*, the clinical variant database; *FH*, familial hypercholesterolemia.

## Discussion

Our study, which used GS data from 2 Taiwanese independent sources, yielded consistent results for the heterozygote frequencies of the CDCT1 genetic variants. We discovered a higher hit rate of more than 1.7% in the 3 databases. Furthermore, the identified variants showed different patterns from those reported by HNP.^1^ FH accounts for more than 60% of the positive cases, compared with approximately 30% observed in the previous study. Additionally, the patterns seemed to vary across different ethnicities worldwide, even among those from Singapore (Southeast Asia)^23^ and Japan (Northern Asia).^25^ The ethnic admixture present in our datasets may have introduced bias into our findings. Although the TWB and NHRI cohorts are predominantly Han Chinese, previous genomic studies have shown that Taiwanese populations include varying degrees of Southern Chinese and other Asian admixture.^6^^,^^10^^,^^22^ Such sub-ancestry differences can influence allele frequencies and may partly explain the observed differences between our results and other Asian datasets. Likewise, shared risk allele profiles between Taiwanese and Singaporeans of Chinese origin likely reflect shared ancestry, whereas variants enriched only in Taiwan may represent local founder effects.

Several studies have demonstrated that only a minority of adults are aware that they carry these pathogenic variants.^29^ Similarly, 42.86% of participants were aware of FH in our study, and only 44% were consuming statins. This gap in awareness may be because of the limitations in the collection and use of family history. These findings are particularly concerning, given that the failure to identify at-risk individuals persists even as the costs of DNA testing decline and the ability to detect pathogenic alleles in genomes improve.

To address these challenges, management guidelines must include regular reanalysis of DNA variants, informed by the most up-to-date curated databases, and ongoing clinical evaluation of screened individuals. Additionally, the availability of updated clinical decision support tools, integration with electronic health records, and regular assessment of the effectiveness, benefits, and potential harms of testing and prevention strategies are essential to improve outcomes.^30^

This data collection and analysis approach has enhanced the reliability of our findings. Our results showed that CDCT1 genetic screening is potentially beneficial and could be used by health systems serving EAS populations. We created the acronym “ISAC”—identification, surveillance, action, cost-effectiveness—to help pinpoint its usefulness. Specifically, the acronym accommodates the following points:

1. CDCT1 genetic screening holds immense potential for identifying a significantly larger number of individuals at high risk for 3 hereditary conditions, thereby enabling early intervention and personalized health care.
2. CDCT1 genetic screening can be used to carry out genetic surveillance of individuals at a younger age, allowing family-led screening.
3. Heterozygotes may then take follow-up actions to reduce the risks associated with a relevant genetic variant.
4. CDCT1 genetic screening is cost-effective when used for medical care and patient management.^31^

Taking the “ISAC” principles, we have evaluated the possibility of screening for CADASIL and HHL in our population. Profiling *NOTCH3* P/LP and biallelic *GJB2* variants can provide individuals who are at-risk with information indicating that they should take caution and undertake regular health examinations/evaluations. Integrating these into the CDCT1 screen of local variants highlights the importance of ethnic-specific considerations in population screening. Although it is technically feasible, the impact of the 2 conditions on the outcomes of the screening is not proven. Further studies are warranted to demonstrate their clinical utility in genetic prediction and early intervention.

Our study faces several limitations. Although the *APOB* NC_000002.12:g.21006289G>A p.(Arg3527Trp) and both *LDLR* variants (NC_000019.10:g.11102741G>A p.(Asp90Asn) and NC_000019.10:g.11116900C>T p.(His583Tyr) demonstrated a strong association with FH in the Taiwanese population, these findings need to be validated in broader populations. The relatively small sample size is another limitation because the consistency was only confirmed by validating our high AF variants with the TWB array data, except for 2 variants, *LDLR* NC_000019.10:g.11116900C>T p.(His583Tyr) and *GJB2* NC_000013.11:g.20189473C>T p.(Val37Ile). Furthermore, effective treatments for CADASIL and HHL are currently unavailable compared with CDCT1 conditions, suggesting potential gaps in translational research. Lastly, establishing genetic counseling and training organizations is challenging and needs to be improved within the clinical and government systems in Taiwan. At-risk heterozygotes must receive appropriate guidance and support from clinicians to undergo interventions that can improve their health. This is particularly true for those with *NOTCH3* NC_000019.10:g.15187315G>A p.(Arg544Cys) and *GJB2* NC_000013.11:g.20189473C>T p.(Val37Ile) because both variants are frequently present in Asian populations. Control of vascular disease risk factors, such as blood pressure, and avoidance of thrombolytic therapy and anticoagulants should be advised for *NOTCH3* NC_000019.10:g.15187315G>A p.(Arg544Cys) heterozygotes. On the other hand, avoidance of noise exposure and using hearing protection are crucial for reducing the risk of hearing loss in *GJB2* NC_000013.11:g.20189473C>T p.(Val37Ile) heterozygotes.

Our study facilitates a comparative analysis of variant frequencies in Taiwan with those of diverse global populations, potentially significantly impacting the health care of the Taiwanese population. Furthermore, we have systematically compared genetic profiles, clinical manifestations, and survey statistics between heterozygotes and non-heterozygotes, elucidating possible associations between phenotypes and heterozygotes. This in-depth analysis seeks to provide a comprehensive understanding of the genetic risks associated with these critical genetic disease conditions, thereby addressing a substantial knowledge gap and eventually enhancing the health outcomes of the Taiwanese population.

## Conflict of Interest

Shih-Feng Tsai and Ralph Kirby are cofounders of two companies, PDC4PM and TIGeR. Both of these companies are involved in the commercial application of whole genome sequencing.
