# Supplementary material for: Implications and ramifications of using the CDC tier 1 genetic screening concept in East Asian populations
Source: Genet Med Open. 2026 Jun 6;4:104410. doi: 10.1016/j.gimo.2026.104410 (PMC13508668; doi:10.1016/j.gimo.2026.104410)
Supplement: Supplementary file 1 [file mmc1.pdf]

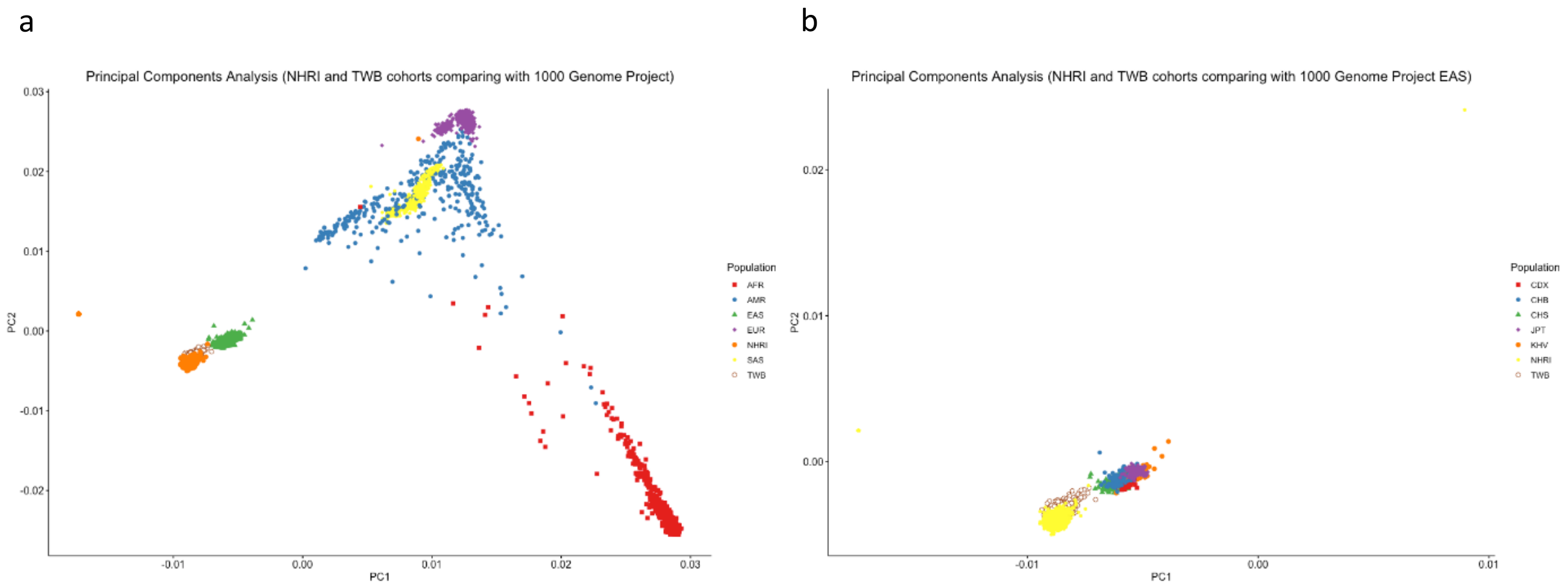

Supplementary Figure 1. Population structure of the NHRI and TWB cohorts. (a) NHRI and TWB participants clustered with the 1000 genomes EAS (East Asian) population. (b) NHRI and TWB participants exhibited a close genetic relationship with the 1000 genomes CHB (Han Chinese in Beijing) and CHS (Han Chinese from Southern China) populations.

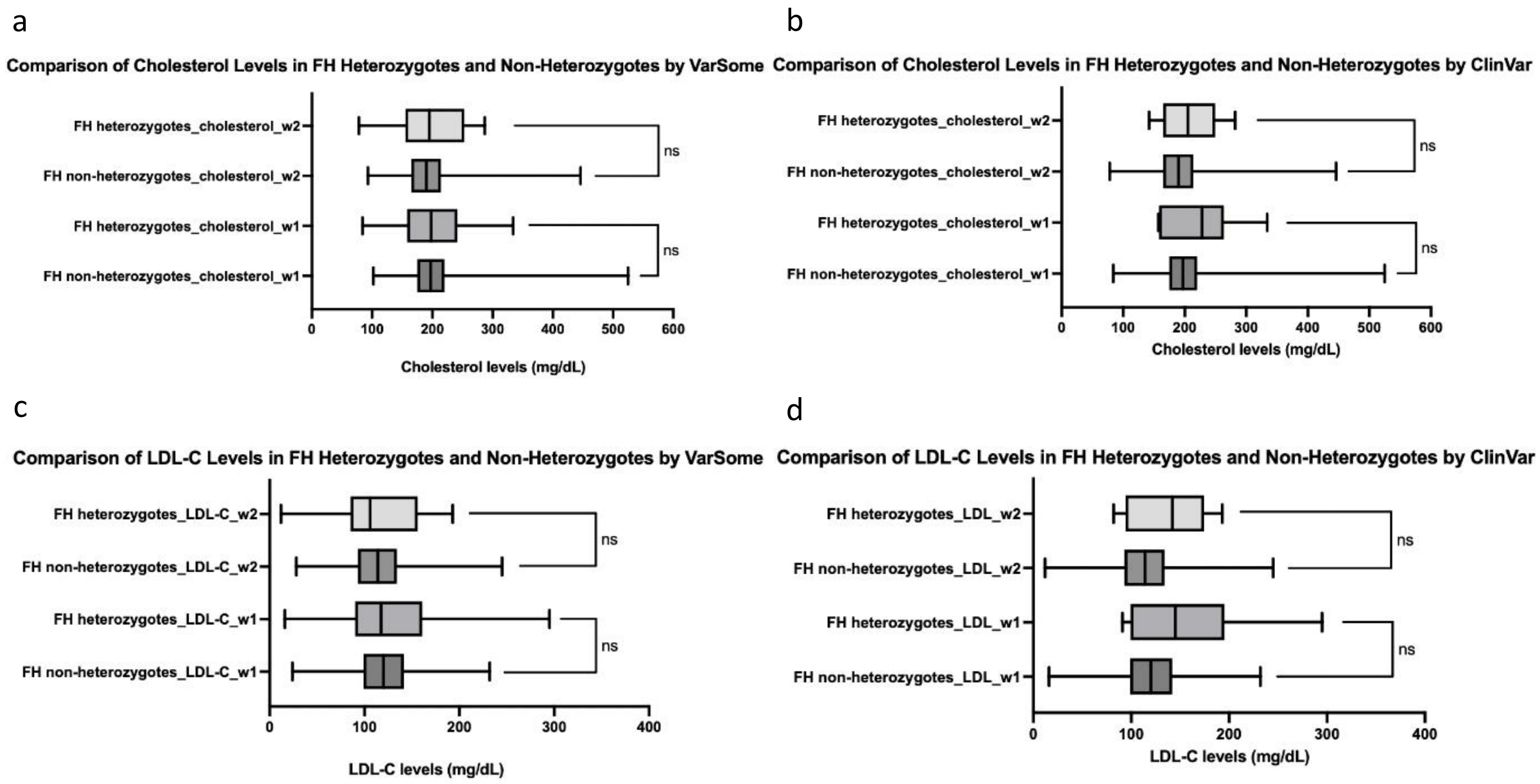

Supplementary Figure 2. The cholesterol and LDL-C levels of individuals with FH heterozygotes and non-heterozygotes, as measured by VarSome and ClinVar, between two waves in the HALST cohort. The cholesterol and LDL-C levels were evaluated in the two waves (wave 1 and wave 2) of the HALST cohort. Individuals who were taking statin medication were not included in the analysis. (a)(b) FH heterozygotes annotated as VarSome and ClinVar were compared to non-heterozygotes in terms of cholesterol levels. (c)(d) FH heterozygotes annotated as VarSome and ClinVar were compared to non- heterozygotes in terms of LDL-C levels.

Supplementary Table 1. Global comparison of allele frequencies with P/LP variants for the CDCT1 genes, *NOTCH3*, and *GJB2*

Genetic Ancestry Group (n = sample counts)	*APOB* NC_000002.12:g.21006289G>A p.(Arg3527Trp)	*LDLR* NC_000019.10:g.11102741G>A p.(Asp90Asn)	*LDLR* NC_000019.10:g.11116900C>T p.(His583Tyr)	*NOTCH3* NC_000019.10:g.15187315G>A p.(Arg544Cys)	*GJB2* NC_000013.11:g.20189473C>T p.(Val37Ile)	*GJB2* NC_000013.11:g.20189349del p.(Leu79fs)	*GJB2* NC_000013.11:g.20189282_20189283del p.(His100fs)
**Taiwanese Cohorts**							
**NHRI cohorts (WGS) (n = 2,473)**	0.0012131	0.0012131	0.0024262	0.0044480	0.0901739	0.0052568	0.0006066
**Taiwan Biobank (WGS) (n = 1,454)**	0.0030949	0.0017194	0.0020633	0.0037827	0.0866575	0.0061898	0.0017194
**TPMI Array (n = 37,790)**	0.0018659	0.0007675	NA	0.0048955	NA	0.0064567	0.0013231
**TWB 1.0 Array (n = 24,674)**	NA	NA	NA	NA	NA	NA	NA
**TWB 2.0 Array (n = 100,277)**	0.0020343	0.0009978	NA	NA	NA	NA	NA
**SG10K**							
**SG10K-Chinese (n = 2,780)**	0.0015200	0.0006374	0.0012870	0.0046221	0.1007026	0.0052905	0.0013499
**SG10K-Malay (n = 903)**	0.0013320	0	0	0.0013391	0.0790610	0	0.0012570
**SG10K-India (n = 1,127)**	0	0	0	0.0012112	0.0062500	0	0
**Total (n = 4,810)**	0.0011480	0.0003715	0.0007541	0.0032187	0.0003715	0.0030842	0.0009966
**TogoVar**							
**TOMMO_54K_JAPAN (WGS) (n = 54,302)**	0.0000092	0	0	0.0000180	0.0099996	0.0074768	0.0003867
**GnomAD**							
**East Asian (n = 22,448)**	0.0005126	0.0003120	0.0004900	0.0014260	0.0448809	0.0066628	0.0009581
**Remaining (n = 31,256)**	0	0.0000320	0.0001120	0.0000800	0.0069287	0.0000960	0
**European (Non-Finnish) (n = 590,031)**	0.0000279	0.0000127	0	0.0000034	0.0008816	0.0000025	0.0000025
**South Asian n = (45,546)**	0.0003843	0.0000109	0.0001757	0.0002306	0.0001537	0.0000110	0.0000110
**Middle Eastern (n = 3,031)**	0.0001650	0	0	0	0.0018158	0	0
**African/African American (n = 37,545)**	0.0000133	0	0	0	0.0007330	0	0
**Admixed American (n = 30,019)**	0	0	0	0.0000167	0.0028485	0	0
**European (Finnish) (n = 32,026)**	0	0	0	0	0.0016555	0	0
**Ashkenazi Jewish (n = 14,804)**	0	0	0	0.0000338	0.0077069	0	0
**Amish (n = 456)**	0	0	0	0	0	0	0
**Total (n = 807,162)**	0.0000576	0.0000198	0.00002788	0.0000595	0.0025228	0.0001914	0.0000291

Supplementary Table 4. Individuals with CADASIL and HHL P/LP variants as identified by ClinVar and VarSome

Studied cohorts	Annotation	*NOTCH3* (%)	*NOTCH3* NC_000019.10:g.15187315G>A p.(Arg544Cys) (%)	*GJB2* (%)	*GJB2* NC_000013.11:g.20189473C>T p.(Val37Ile) (homozygous)(%)	*GJB2* NC_000013.11:g.20189473C>T p.(Val37Ile) (homozygous+heterozygous)(%)
**Individuals with P/LP variants in TWB**						
**TWB cohorts (n = 1,454)**	**ClinVar**	11 (0.75%)	11 (0.75%)	267 (18.36%)	10 (0.69%)	242 (16.64%)
	**VarSome**	16 (1.10%)	11 (0.75%)	271 (18.64%)	10 (0.69%)	242 (16.64%)
**Male (n = 717)**	**ClinVar**	7 (0.98%)	7 (0.98%)	131 (18.27%)	3 (0.42%)	120 (16.74%)
	**VarSome**	10 (1.39%)	7 (0.98%)	132 (18.41%)	3 (0.42%)	120 (16.74%)
**Female (n = 737)**	**ClinVar**	4 (0.54%)	4 (0.54%)	137 (18.59%)	7 (0.95%)	122 (15.60%)
	**VarSome**	6 (0.81%)	4 (0.54%)	139 (18.76%)	7 (0.95%)	122 (15.60%)
**Individuals with P/LP variants in NHRI cohorts**						
**NHRI cohorts (n = 2,473)**	**ClinVar**	23 (0.93%)	22 (0.89%)	483 (19.53%)	25 (1.01%)	421 (17.02%)
	**VarSome**	28 (1.13%)	22 (0.89%)	494 (19.98%)	25 (1.01%)	421 (17.02%)
**Male (n = 1,270)**	**ClinVar**	11 (0.87%)	11 (0.87%)	246 (19.37%)	16 (1.26%)	210 (16.53%)
	**VarSome**	14 (1.10%)	11 (0.87%)	250 (19.69%)	16 (1.26%)	210 (16.53%)
**Female (n = 1,203)**	**ClinVar**	12 (1.00%)	11 (0.91%)	237 (19.70%)	9 (0.75%)	211 (17.54%)
	**VarSome**	14 (1.16%)	11 (0.91%)	244 (20.28%)	9 (0.75%)	211 (17.54%)
**Individuals with P/LP variants in HALST[a] cohort**						
**HALST[1] (n = 1,016)**	**ClinVar**	7 (0.69%)	7 (0.69%)	208 (20.47%)	7 (0.69%)	190 (18.70%)
	**VarSome**	10 (0.98%)	7 (0.69%)	211 (20.77%)	7 (0.69%)	190 (18.70%)
**Male (n = 443)**	**ClinVar**	2 (0.45%)	2 (0.45%)	87 (19.64%)	4 (0.90%)	77 (17.38%)
	**VarSome**	3 (0.68%)	2 (0.45%)	88 (19.86%)	4 (0.90%)	77 (17.38%)
**Female (n = 573)**	**ClinVar**	5 (0.87%)	5 (0.87%)	121 (21.12%)	3 (0.52%)	113 (19.72%)
	**VarSome**	7 (1.22%)	5 (0.87%)	123 (21.47%)	3 (0.52%)	113 (19.72%)

[a]HALST: Healthy Aging Longitudinal Study in Taiwan

Supplementary Table 5. Statistical analysis of allele frequencies: Taiwanese cohorts vs. four populations from worldwide databases

Variant (Taiwanese AF)	Cohort / dataset	Odds Ratio (OR) (95% Confidence Intervals)	P-value (Fisher's Exact Test)
APOB **NC_000002.12:g.21006289G>A p.(Arg3527Trp)** (p=0.00203)	SG10K Chinese	1.26 (0.64 - 2.50)	0.591
	SG10K Malay	1.35 (0.51 - 3.57)	0.634
	TogoVar Japan	143.14 (26.78 - 765.08)	< 0.001
	gnomAD East Asian	3.77 (1.99 - 7.17)	< 0.001
LDLR **NC_000019.10:g.11102741G>A p.(Asp90Asn)** (p=0.00101)	SG10K Chinese	2.15 (0.85 – 5.39)	0.101
	SG10K Malay	10.88 (0.64 - 184.7)	0.021
	TogoVar Japan	318.47 (18.76 - 5405.06)	< 0.001
	gnomAD East Asian	4.54 (2.09 - 9.84)	< 0.001
LDLR **NC_000019.10:g.11116900C>T p.(His583Tyr)** (p=0.00229)	SG10K Chinese	1.77 (0.89 – 3.52)	0.109
	SG10K Malay	17.17 (1.03 - 285)	0.002
	TogoVar Japan	512.78 (30.9 - 8510.01)	< 0.001
	gnomAD East Asian	4.71 (2.54 - 8.71)	< 0.001
NOTCH3 **NC_000019.10:g.15187315G>A p.(Arg544Cys)** (p=0.004202)	SG10K Chinese	0.91 (0.59 – 1.41)	0.74
	SG10K Malay	2.9 (1.18 - 7.16)	0.014
	TogoVar Japan	186.06 (51.46 - 672.7)	< 0.001
	gnomAD East Asian	2.98 (1.96 - 4.52)	< 0.001
GJB2 **NC_000013.11:g.20189473C>T p.(Val37Ile)** (p=0.088872)	SG10K Chinese	0.87 (0.79 – 0.96)	0.006
	SG10K Malay	1.14 (0.98 - 1.31)	0.081
	TogoVar Japan	9.66 (8.76 - 10.65)	< 0.001
	gnomAD East Asian	2.08 (1.9 - 2.27)	< 0.001
GJB2 **NC_000013.11:g.20189349del p.(Leu79fs)** (p=0.005602)	SG10K Chinese	1.06 (0.72 – 1.57)	0.765
	SG10K Malay	42.6 (2.62 - 691.95)	< 0.001
	TogoVar Japan	0.76 (0.56 - 1.02)	0.064
	gnomAD East Asian	0.85 (0.62 - 1.16)	0.322
GJB2 **NC_000013.11:g.20189282_20189283del p.(His100fs)** (p=0.001019)	SG10K Chinese	0.83 (0.36 – 1.94)	0.672
	SG10K Malay	0.73 (0.25 - 2.13)	0.767
	TogoVar Japan	2.77 (1.32 - 5.78)	0.018
	gnomAD East Asian	1.12 (0.54 - 2.33)	0.844

Supplementary Table 6. Demographics, clinical characteristics, and survey data of FH heterozygotes in the TWB cohort (n = 1,400)						
**Variables**	**Group 1: FH_heterozygotes (VarSome), N = 28**	**Group 2: FH_non-heterozygotes (VarSome), N = 1,372**	**Group 3: FH_heterozygotes (ClinVar), N = 21**	**Group 4: FH_non-heterozygotes (ClinVar), N = 1,379**	**P value (Group 1 vs 2)**	**P value (Group 3 vs 4)**
**Age**	51.6 ± 11.4	52.2 ± 11.6	51.1 ± 12.2	52.2 ± 11.5	ns	ns
**Sex, M/F (F %)**	13/15 (53.6%)	684/688 (50.1%)	9/12 (57.1%)	688/691 (50.1%)	ns	ns
**BMI (kg/m^2)**	24.2 ± 4.6	24.5 ± 3.7	23.4 ± 3.2	24.5 ± 3.7	ns	ns
**Valve heart disease (%)**	3 (10.7%)	67 (4.9%)	3 (14.3%)	67 (4.6%)	ns	< 0.05
**Coronary artery disease (%)**	2 (7.1%)	32 (2.3%)	2 (9.5%)	32 (2.3%)	ns	< 0.05
**Arrhythmia (%)**	2 (9%)	95 (6.9%)	1 (4.8%)	96 (7.0%)	ns	ns
**Cardiomyopathy (%)**	2 (9%)	12 (0.9%)	1 (4.8%)	13 (0.9%)	< 0.001	ns
**Congenital heart disease (%)**	0 (0%)	2 (0.1%)	0 (0%)	2 (0.1%)	ns	ns
**Hypertension (%)**	5 (17.9%)	226 (16.5%)	3 (14.3%)	228 (16.5%)	ns	ns
**Apoploxia (%)**	0 (0%)	15 (1.1%)	0 (0%)	15 (1.1%)	ns	ns
**Hyperlipidemia (%)**	11 (39.3%)	138 (10.1%)	9 (42.9%)	140 (10.2%)	$< 10^{-6}$	$< 10^{-5}$
**TC (mg/dL)**	204.5 ± 54.2	174.3 ± 34.2	208.8 ± 56.8	174.4 ± 34.3	< 0.001	< 0.001
**HDL-C (mg/dL)**	52 ± 13.0	53.6 ± 13.7	51.7 ± 12.0	53.6 ± 13.7	ns	ns
**LDL-C (mg/dL)**	152.4 ± 49.3	120.7 ± 31.2	157.1 ± 51.6	120.8 ± 31.3	< 0.001	< 0.001

Supplementary Table 7. Demographics, clinical characteristics, and survey data of HBOC heterozygotes in the TWB cohort (n = 1,400)						
**Variables**	**Group 1: HBOC_heterozygotes (VarSome), N = 18**	**Group 2: HBOC_non-heterozygotes (VarSome), N = 1,382**	**Group 3: HBOC_heterozygotes (ClinVar), N = 5**	**Group 4: HBOC_heterozygotes (ClinVar), N = 1,395**	**P value (Group 1 vs 2)**	**P value (Group 3 vs 4)**
**Age**	51.6 ± 11.8	52.2 ± 11.6	50.8 ± 13.0	52.2 ± 11.6	ns	ns
**Sex, M/F (F %)**	7/11 (61.1%)	690/692 (50.1%)	4/1 (20%)	693/702 (45.5%)	ns	ns
**BMI (kg/m^2)**	25.3 ± 4.1	24.5 ± 3.7	26.7 ± 5.1	25.1 ± 3.7	ns	ns
**Prostate Cancer (%)**	0 (0%)	0 (0%)	0 (0%)	0 (0%)	ns	ns
**Breast Cancer (%)**	0 (0%)	5 (0.7% in female)	0 (0%)	5 (0.7% in female)	ns	ns
**Ovarian Cancer (%)**	0 (0%)	0 (0% in female)	0 (0%)	0 (0%)	ns	ns
**Uterine Cancer (%)**	0 (0%)	1 (0.1% in female)	0 (0%)	1 (0.1% in female)	ns	ns
**Cervical Cancer (%)**	0 (0%)	1 (0.1% in female)	0 (0%)	1 (0.1% in female)	ns	ns
**Myoma (%)**	3 (27.3% in female)	159 (23% in female)	0 (0%)	162 (23.1% in female)	ns	ns
**Ovarian Cyst (%)**	0 (0%)	61 (8.8% in female)	0 (0%)	61 (8.7% in female)	ns	ns
**Endometrisis (%)**	0 (0%)	54 (7.8% in female)	0 (0%)	54 (7.7% in female)	ns	ns
**Cervical Polyp (%)**	0 (0%)	33 (4.8% in female)	0 (0%)	33 (4.7% in female)	ns	ns

Supplementary Table 8. Demographics, clinical characteristics, and survey data of LS heterozygotes in the TWB cohort (n = 1,400)						
**Variables**	**Group 1: LS_heterozygotes (VarSome), N = 31**	**Group 2: LS_non-heterozygotes (VarSome), N = 1,369**	**Group 3: LS_heterozygotes (ClinVar), N = 8**	**Group 4: LS_non-heterozygotes (ClinVar), N = 1,392**	**P value (Group 1 vs 2)**	**P value (Group 3 vs 4)**
**Age (year range)**	54.4 ± 9.9	52.2 ± 11.6	56.1 ± 8.5	52.2 ± 11.6	ns	ns
**Sex, M/F (F %)**	14/17 (54.8%)	683/686 (50.1%)	3/5 (62.5%)	694/698 (50.1%)	ns	ns
**BMI (kg/m^2)**	23.6 ± 3.6	24.5 ± 3.7	23.3 ± 3.9	24.5 ± 3.7	ns	ns
**Gastric Cancer (%)**	0 (0%)	0 (0%)	0 (0%)	0 (0%)	ns	ns
**Colorectal Cancer (%)**	0 (0%)	3 (0.2%)	0 (0%)	3 (0.2%)	ns	ns
**Ovarian Cancer (%)**	0 (0%)	0 (0%)	0 (0%)	0 (0%)	ns	ns
**Uterine Cancer (%)**	0 (0%)	1 (0.1% in female)	0 (0%)	1 (0.1% in female)	ns	ns
**Cervical Cancer (%)**	0 (0%)	1 (0.1% in female)	0 (0%)	1 (0.1% in female)	ns	ns
**Myoma (%)**	5 (29.4% in female)	157 (22.9% in female)	1 (20% in female)	161 (23.1% in female)	ns	ns
**Ovarian Cyst (%)**	1 (5.9% in female)	60 (8.7% in female)	0 (0%)	61 (8.7% in female)	ns	ns
**Endometrisis (%)**	3 (17.6% in female)	51 (7.4% in female)	1 (20% in female)	53 (7.6% in female)	ns	ns
**Cervical Polyp (%)**	0 (0%)	33 (4.8% in female)	0 (0%)	33 (4.7% in female)	ns	ns

Supplementary Table 9. The usage of statin or other cholesterol-related drugs of FH heterozygotes in the HALST cohort					
**Annotated tools**	**FH heterozygotes**	**Drug using status in W1 (%)**	**Drug using status in W2 (%)**	**Increment**	**FH heterozygotes without using drugs (%)**
**VarSome**	19	7(37%)	7(37%)	+0	12 (63%)
**ClinVar**	9	3(33%)	4(44%)	+1	5 (56%)
